# Therapeutic Effects of Berberine Hydrochloride on Stress-Induced Diarrhea-Predominant Irritable Bowel Syndrome Rats by Inhibiting Neurotransmission in Colonic Smooth Muscle

**DOI:** 10.3389/fphar.2021.596686

**Published:** 2021-09-14

**Authors:** Yulin Lu, Jingjing Huang, Yao Zhang, Zitong Huang, Weiming Yan, Tianran Zhou, Zhesheng Wang, Lu Liao, Hongying Cao, Bo Tan

**Affiliations:** ^1^ Research Center for Integrative Medicine, School of Basic Medical Sciences, Guangzhou University of Chinese Medicine, Guangzhou, China; ^2^ School of Chinese Materia Medica, Guangzhou University of Chinese Medicine, Guangzhou, China; ^3^ The Third Clinical Medical College, Guangzhou University of Chinese Medicine, Guangzhou, China; ^4^ The Fourth Clinical Medical College, Guangzhou University of Chinese Medicine, Shenzhen, China

**Keywords:** berberine hydrochloride, diarrhea-predominant irritable bowel syndrome, colonic longitudinal smooth muscles, inhibitory effects, neurotransmission

## Abstract

The etiology of diarrhea-predominant irritable bowel syndrome (IBS-D) is complicated and closely related to neurotransmission in the gastrointestinal (GI) tract. Developing new strategies for treating this disease is a major challenge for IBS-D research. Berberine hydrochloride (BBH), the derivative of berberine, is a herbal constituent used to treat IBS. Previous studies have shown that BBH has potential anti-inflammatory, antibacterial, analgesic, and antidiarrheal effects and a wide range of biological activities, especially in regulating the release of some neurotransmitters. A modified IBS-D rat model induced by chronic restraint stress was used in all experiments to study the effects of BBH on the GI tract. This study measured the abdominal withdrawal reflex (AWR) response to graded colorectal distention (CRD; 20, 40, 60, and 80 mmHg) and observed the fecal areas of stress-induced IBS-D model. Experiments were conducted using organ bath techniques, which were performed *in vitro* using strips of colonic longitudinal smooth muscle. Inhibitory and excitatory neurotransmitter agents were added to each organ bath to observe contractile responses on the strips and the treatment effect exerted by BBH. The IBS-D rat model was successfully induced by chronic restraint stress, which resulted in an increased defecation frequency and visceral hypersensitivity similar to that of humans. BBH could reduce 4-h fecal areas and AWR response to CRD in IBS-D. The stress-induced IBS-D model showed upregulated colonic mRNA expression levels of 5-hydroxytryptamine-3A receptor and downregulated expression levels of neuronal nitric oxide synthase. Meanwhile, BBH could reverse this outcome. The responses of substances that regulate the contraction induced by related neurotransmission in the longitudinal smooth muscle of IBS-D colon (including the agonist of acetylcholine, carbachol; NOS inhibitor, L-NAME; and P2Y_1_ receptor antagonist, MRS2500) can be inhibited by BBH. In summary, BBH promotes defecation frequency and visceral hypersensitivity in IBS-D and exerts inhibitory effects on contractile responses in colonic longitudinal smooth muscle. Thus, BBH may represent a new therapeutic approach for treating IBS-D.

## Introduction

Irritable bowel syndrome (IBS) is a prevalent gastrointestinal (GI) disease characterized by abdominal pain and change in bowel habits. IBS has affected 11.2% of the general population worldwide (Ford, 2020). Many factors, such as heredity, diet, mentality, social culture, and inflammatory substances, influence its onset. IBS incurs a heavy economic and social burden on an individual, as well as the society. The Rome Ⅳ standard is the latest diagnostic criteria used to categorize patients with IBS into four types. Diarrhea-predominant IBS(IBS-D) is a subtype associated with rectal urgency, increased defecation frequency, abdominal bloating, and loose to watery stools and accounts for 23.4% of patients with IBS (Clarke et al., 2009). Currently, drugs targeting neurotransmitter receptors, such as loperamide, iludoline, alosetron and some antidepressants, are used to treat patients with IBS-D (Lacy, 2016). However, a standard treatment algorithm has not been established yet for IBS-D (Corsetti and Whorwell, 2017).

Current research has shown that GI motility and mucus secretion, as well as the sensitivity of the GI tract to mechanical or chemical sensory stimuli, can be altered by abnormalities in the enteric nervous system (ENS) (Gwynne and Bornstein, 2007; Peiris et al., 2017). The intestinal plexus is involved in regulating intestinal peristalsis. The study on the rat model of IBS induced by neonate maternal separation shows that the increase of colonic motility is related to the up-regulation of L-type calcium (Zhang et al., 2010). Other results suggest that the transient receptor potential vanilloid type-1 (TRPV1 or VR1) immunoreactive nerve fibers in colonic biopsies from patients with IBS can be activated by intestinal chemical mediators and release neurotransmitters such as substance P and calcitonin gene-related peptide. Moreover, the increase of TRPV1 nerve fibers may be related to visceral hypersensitivity and pain in IBS (Akbar et al., 2008). In short, the changes in GI motility involved in IBS-D are closely related to the mechanical relaxation and contraction of GI smooth muscle, which are jointly regulated by transmitters released in ENS, especially in the intermuscular plexus.

As an important non-adrenergic and non-cholinergic neurotransmitter regulating smooth muscle contraction, nitric oxide (NO) is widely distributed in the GI tract and has important biological functions, especially in the regulation of GI motility and visceral sensation (Konturek and Konturek, 1995). In addition, the effects of NO-mediated antinociception and anti-diarrhea in the IBS-D rat model have been confirmed (Paragomi et al., 2014). Intestinal peristalsis depends on the contraction of smooth muscle and is regulated by interstitial cells of Cajal (ICC) in the GI tract (Lammers et al., 2011). NO is produced from arginine in a reaction catalyzed in the intestine by NO synthases (NOS), in which neuronal NOS (nNOS) plays an important role in regulating GI peristalsis, and the distribution of nNOS immunoreactive positive products in GI mucous epithelial cells, myenteric plexus and submucosal ganglion cells has been confirmed (Ravella et al., 2013; Tomuschat et al., 2017). NO is an unstable lipid-soluble small molecular compound, which is synthesized and diffused to the target ICC by diffusion, activating calcium pumps dependent on cyclic guanosine monophosphate (cGMP) protein kinase, preventing calcium ion influx, or promoting calcium ion transmembrane transport out of cells. It also participates in cell-to-cell information transmission (Toda and Herman, 2005; Cain and Snutch, 2011). Therefore, as a messenger between nNOS positive neurons and GI smooth muscle cells, the increase of NO reduces the influx of calcium ions and directly promotes the relaxation of smooth muscle.

The neurohormone 5-hydroxytryptamine (5-HT or serotonin) is considered to be an important intestinal neurotransmitter, about 95% of which exist in the intestinal tract in the human body. It plays an important role in regulating GI motility and secretion, coordinating reflexes, and regulating sensory information in and out of the intestine. Within the bowel, serotonin is synthesized by the enterochromaffin (EC) subtype of enteroendocrine cells and by serotonergic neurons in the myenteric plexus (Gershon and Tack, 2007). 5-HT_3_ receptors are widely distributed in peripheral and central nervous tissues, through which 5-HT acts to excite enteric neurons (Derkach et al., 1989). Current studies have confirmed that 5-HT_3_ receptor antagonists can effectively inhibit intestinal urgency, prolong intestinal and colorectal conduction and relieve the symptoms of IBS-D (Oh et al., 2001).

The concept of purinergic signaling stems from studies that were designed to identify the non-adrenergic, non-cholinergic (NANC) inhibitory neurotransmitter in the gut (Burnstock et al., 1972). Purine receptors are broadly classified as P1 for nucleosides (adenosine) and P2 for nucleotides (ATP). They can be subdivided into adenosine (A_1_, A_2A_, A_2B_, A_3_), P2X_1-7,_ and P2Y_1,2,4,6,11–14_. Most of these receptors are expressed in the GI tract where they are known to be involved in the physiological regulation of gut reflexes in animal models. Endogenous purines are critical regulators of neurotransmission in the human ENS acting at P2X_1_, P2X_2_, and P2X_3_ channels, as well as inhibitory P2Y or A_3_ receptors. These purinergic receptors and signaling pathways are essential as they are potential therapeutic targets for inflammatory bowel diseases (IBDs), IBS and diarrheal disorders (Liñán-Rico et al., 2015).

To sum up, neurotransmission may contribute to the homeostatic maintenance of the ENS and play an important role in the pathogenesis of IBS-D.

**FIGURE 1:**
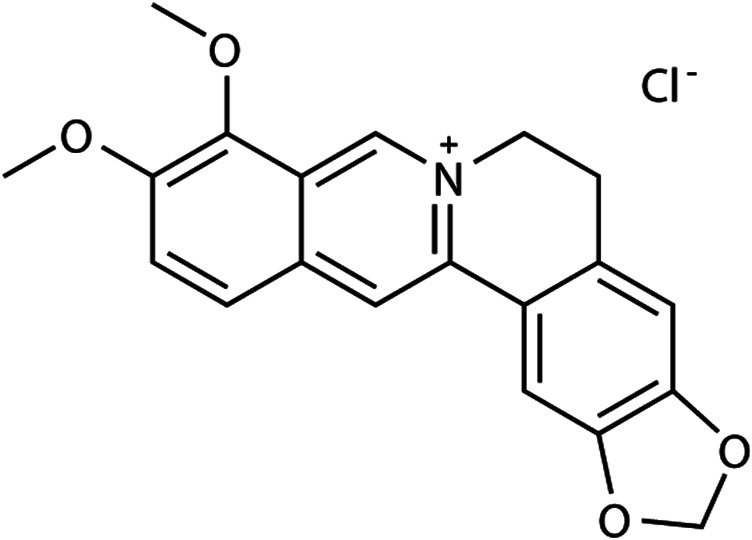
Structure of BBH.

*Rhizoma coptidis* is a traditional Chinese herb widely used as a remedy for GI diseases, particularly against diarrhea (Chen et al., 2015). Berberine hydrochloride (BBH; its structure is shown in Figure 1), a berberine derivative, is a benzylisoquinoline alkaloid found in *Rhizoma coptidis* and has potential therapeutic effects as an antiemetic, antimicrobial, anti-inflammatory, and antinociceptive substance. BBH may play a role in the treatment of diarrhea (Cheng et al., 2009). A systematic review and meta-analysis about children and adults demonstrated that berberine was generally effective in improving clinical cure rates and shortening the duration of diarrhea (Yu et al., 2020). Studies on colitis models induced by intracolonic instillation have shown that BBH can significantly reduce visceral sensitivity and the frequency of defecation in rats, which may be achieved through NO-mediation (Tang et al., 2013). Furthermore, BBH inhibits GI motility in rodents and is closely related to the endogenous opioid system (Chen et al., 2015). These results implied that BBH might exert a therapeutic effect on IBS-D. However, its mechanism has not been fully clarified to date, and no further studies have reported the role of BBH in regulating the pharmacology of digestive system-related neurotransmitters. The present study mainly explores how the neurotransmission of the GI tract affects IBS-D and whether BBH can treat IBS-D in rats through GI neurotransmitters.

## Materials and Methods

### Chemicals and Reagents

The Krebs solution used in this study was composed of 120 mmol/L NaCl, 5.9 mmol/L KCl, 25 mmol/L NaHCO_3_, 1.2 mmol/L Na_2_HPO_4_·12H_2_O, 1.2 mmol/L MgCl_2_·6H_2_O, 2.5 mmol/L CaCl_2_, and 11.5 mmol/L dextrose. The following materials were used in the study: BBH (Aladdin, China), pinaverium bromide (30 mg/kg, Abbott Products SAS, France), TRIzol reagent (Thermo Fisher, United States), RevertAid First-Strand cDNA Synthesis Kit (Thermo Fisher, United States), SuperReal PreMix Plus [SYBR Green; a special reagent for quantitative real-time polymerase chain reaction (qRT-PCR) by chimeric fluorescence method; Tiangen, China], dimethyl sulfoxide (DMSO; <0.2%; Thermo, United States; used as a medium to dissolve BBH in all experiments), tetrodotoxin (TTX; 1 μmol/L, Thermo Fisher, United States), carbachol (CCh; 1 × 10^−9^–1 × 10^–5^ mol/L; Sigma-Aldrich, United States), N^ω^-nitro-L-arginine methyl ester hydrochloride (L-NAME; 10 μmol/L; Sigma-Aldrich, United States), α,β-methyleneadenosine 5′-triphosphate trisodium salt (α,β-MeATP; 100 μmol/L; Tocris Bioscience, United Kingdom), and (1R*,2S*)-4-[2-iodo-6-(methylamino)-9H-purin-9-yl]-2-(phosphonooxy)bicyclo [3.1.0]hexane-1-methanol dihydrogen phosphate ester tetraammonium salt (MRS2500; 1 μmol/L; Tocris Bioscience, United Kingdom). All reagents were dissolved in distilled water except for KCl, which was distilled in Krebs solution.

### Animal Preparation

Male Sprague–Dawley (SD) rats (aged 4 weeks and weighing 100 ± 10 g) were purchased and fed in specific pathogen-free animal house in strict accordance with the Guide to Animal Use and Care published by the Research Center for Laboratory Animals (Guangzhou University of Chinese Medicine, China). The rats were maintained in adaptable circumstance with 12 h/12 h light/dark cycle, 20–25°C environmental temperature, and 50–70% humidity. The study was reviewed and approved by the Institutional Review Board of Guangzhou University of Chinese Medicine. All procedures used in this study that involved animals were reviewed and approved by the Institutional Animal Care and Use Committee of Guangzhou University of Chinese Medicine (IACUC protocol number: S2018020).

### Stress-Induced IBS-D Rat Model and Administration

The IBS-D rat models used in all experiments were induced by “chronic restraint stress” as described in our previous study (Zhou R. T. et al., 2018) to investigate the effects of BBH on the GI tract. The pathogenesis of IBS remains speculative and may be multifactorial and thus may cause trouble to the research on the modeling mechanism and corresponding drugs under multiple factors. We were concerned that the model induced by single-factor stimulation can stably simulate the symptoms of diarrhea or increased defecation in humans with high visceral sensitivity while excluding the organic lesions of IBS-D. In our previous studies (Zhou TR. et al., 2018), we showed that the abnormalities of neurotransmission in this model occurred in the colon but not in the jejunum.

All the male rats were randomly divided into two groups (50 rats in the model group and 10 rats in the control group) after 7 days of adaptation. The rats in the model group were subjected to restraint stress using an elastic bandage to restrict the movement of the upper body and forelimbs and then anesthetized with ether. Their foreshoulders, upper forelimbs, and thoracic trunk were wrapped in elastic bandage for 2 h each day for 14 days to produce a steady and consistent amount of stimulation to restrict but not prevent movement. The control animals were anesthetized with ether but not restrained. The rats subjected to chronic restraint stress were randomly divided into five groups, namely, the model group (without any drug treatment), the positive drug group (30 mg/kg pinaverium bromide), and three BBH groups with different BBH doses (25, 50, and 100 mg/kg). Then, they were subjected to pharmacological treatment for 14 days. The drugs were given by intragastric administration according to body mass volume and did not exceed 10 ml/kg once a day. The control and model groups were treated with the same volume of normal saline. Specific BBH doses were based on previous studies (Tang et al., 2013; Gong et al., 2014).

### Measurements of Fecal Area and Visceral Hypersensitivity

**FIGURE 2:**
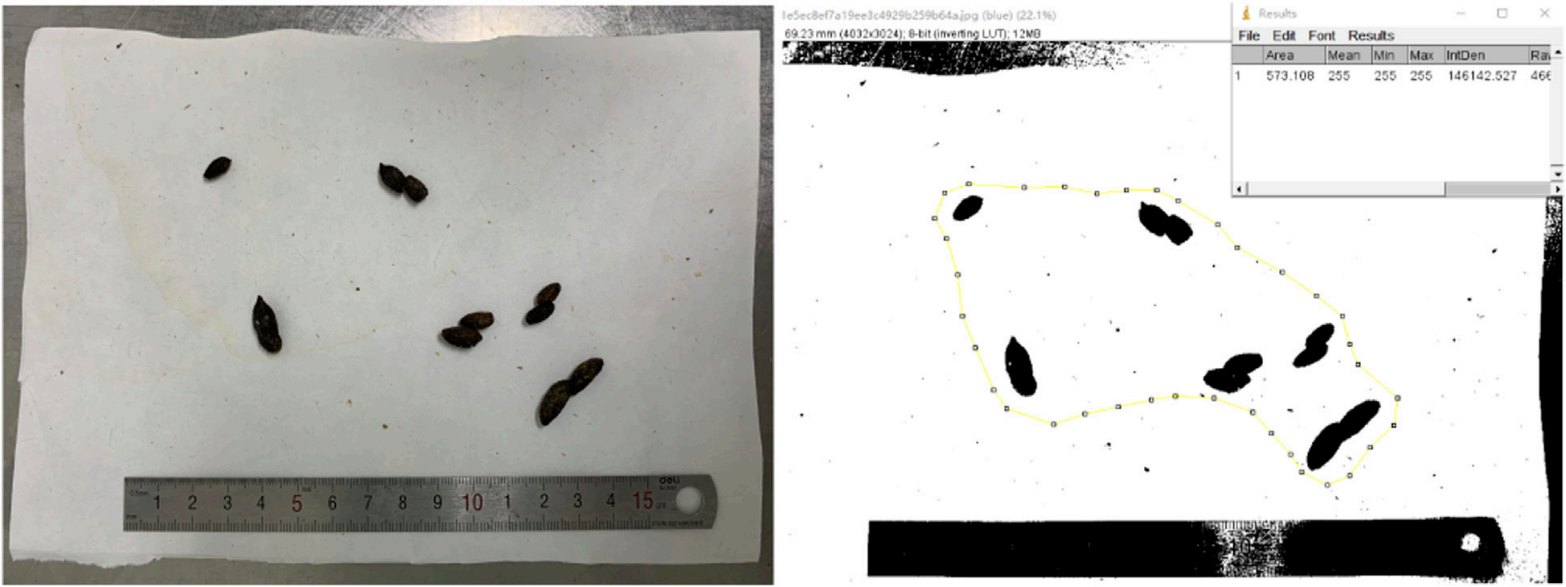
Graph of the excrement before and after ImageJ analysis.

We evaluated the rat model by measuring fecal areas using ImageJ software (Schneider et al., 2012) and observing visceral hypersensitivity responses to colorectal distention (CRD) (Al-Chaer et al., 2000). Rat fecal areas in 4 h were recorded by capturing vertical photos after fixing the height and shooting mode of the digital camera (Bai et al., 2009). The graph of the excrement before and after ImageJ analysis is shown in Figure 2. Behavioral responses to CRD were then assessed in all groups by measuring the abdominal withdrawal reflex (AWR) using a semiquantitative score. A catheter (8Fr) was inserted into the descending colon of the rat anesthetized with ether and secured by taping the attached tubing to the rat’s tail with the outer end of the balloon at about 1 cm from the anus. Then, the catheter was connected with a sphygmomanometer through a three-way valve. AWR measurement consisted of a visual observation of animal response to graded CRD (20, 40, 60, and 80 mmHg) by blinded observers and the assignment of an AWR score: 0, no behavioral response to CRD; 1, brief head movement followed by immobility; 2, contraction of abdominal muscles; 3, lifting of abdomen; 4, body arching and lifting of pelvic structures. The rats were given CRD for 20 s every 4 min, and the expansion was repeated five times for each intensity in the threshold intensity of CRD and AWR measurement.

### Total RNA Extraction and qRT-PCR

The expression of 5-hydroxytryptamine-3A receptor (5-HT_3A_R), nNOS, dynein light chain 8 (DLC8 or LC8), and solute carrier family 17 member 9 (SLC17A9) at the mRNA level were evaluated by qRT-PCR. Briefly, colonic tissues (50–100 mg) were eviscerated from SD rats euthanized by CO_2_ asphyxiation to prepare homogenates after the end of the last administration. Total RNA was isolated from the colonic tissues using TRIzol solution in accordance with the manufacturer’s instructions and treated with RNase-free DNase. Reverse transcription was performed in a reaction system with RevertAid First-Strand cDNA Synthesis Kit. qRT-PCR reactions (20 μl) were set up by the addition of 8 μl of primer mix (containing 10 μM reverse and forward primers), 2 μl of diluted cDNA template, and 10 μl of TaqMan Gene Expression Master Mix (2× SuperReal PreMix Plus). qRT-PCR was performed with a Bio-Rad qRT-PCR System. The relative abundance of glyceraldehyde-3-phosphate dehydrogenase mRNA was used to normalize the levels of mRNAs of interest. All primers used for qPCR are listed in Table 1.

**TABLE 1:** Primers used for qRT-PCR in this study.

Proteins	Upstream primer sequence (5′–3′)	Downstream primer sequence (3′–5′)	Quantity of product (bp)
GAPDH	TGG​CCT​CCA​AGG​AGT​AAG​AAA​C	TGG​AAT​TGT​GAG​GGA​GAT​GCT​C	109
5-HT_3A_R	AGA​ACT​GCT​CTC​TGA​CCT​TCA​C	TTG​TCC​GAC​CTC​ACT​TCT​TCT​G	94
nNOS	TGG​CAA​ATC​GCA​CAA​AGC​TC	TAC​GGG​TTG​TTA​AGG​ACC​ACA​G	102
LC8	TAG​CAT​GGA​CTG​TGC​CAA​AC	TGG​TGC​CCT​GTA​CAA​AAC​AC	140
SLC17A9	TTC​CTG​CCA​GTT​TGT​TCA​GC	AAT​GCT​TGA​CAG​ACC​AAG​GC	113

### Preparation of the Colonic Longitudinal Smooth Muscle Strips

Strips of colonic longitudinal smooth muscle were used in *in vitro* experiments through standard organ bath techniques, and the mechanical activity of the muscle strips was recorded as changes in isometric force. The strips of colonic longitudinal smooth muscles (10 mm long), eviscerated from euthanized SD rats, were cut and suspended in a culture dish. The sample strips were equilibrated in organ baths (the volume of each bath is 5 ml; LE13206, Harvard Apparatus Corporation, United States), which were continuously perfused with Krebs solution that was bubbled with a mixture of 5% CO_2_ and 95% O_2_ (pH 7.4) and maintained at 37°C. The strips were suspended in the direction of the longitudinal muscle. One end of each tissue was connected to a fixed hook, and the other end was connected to a flexible hook. The surgical suture was connected to a force transducer (BR4740-ISO510A, Harvard Apparatus Corporation, United States), which was connected to a computer through an amplifier to record mechanical activity. Changes in tension due to the relaxation or contraction of muscles were recorded through an analog-to-digital board connected to a computer (PL33508, ADInstruments, Powerlab Corporation, Australia). Data were digitized using LabChart Reader (Harvard Apparatus Corporation, United States; LabChart, New Zealand) by a computer. Electrical-field stimulation (EFS; 40 V, 2–30 Hz, 0.5 ms pulse duration, 10 s) was applied *via* two platinum ring electrodes, which were connected to a stimulator (LE12406, Harvard Apparatus Corporation, United States) and attached to each strip. The tissues were given basal tension at 1.4 g after 0.5 h of non-tension adaptive treatment and equilibrated for approximately 1 h prior to the commencement of the experiments. Each colonic tissue was weighed and recorded after blotting on filter paper at the end of the experiments.

### Measurement of the Contraction of Colonic Longitudinal Smooth Muscles

After washing in organ bath and equilibration, BBH was added to each bath in a sequence of concentrations (1.5625, 3.125, 6.25, 12.5, 25, 50, 100 and 200 μmol/L) to observe its effect on the spontaneous contraction of colonic longitudinal smooth muscle. Then, the 50% effective concentration (EC_50_) of BBH was calculated to determine its most effective concentration. In addition, the contractile responses of the BBH- and DMSO-treated groups were compared.

CCh (1 × 10^−9^–1 × 10^–5^ mol/L) was added to the organ bath, and the contractile responses of the longitudinal muscular strips of the control and model groups were compared. After the contraction induced by CCh reached a plateau, the muscle strips were washed with Krebs solution three times and equilibrated for 1 h before the next experiment.

The tissues were stimulated by EFS to induce neuron-mediated contraction, and the frequency–response curve was obtained at an interval of 3 min. Substances able to modulate neurotransmission pathways, including TTX (1 μmol/L), L-NAME (10 μmol/L), α,β-MeATP (100 μmol/L), and MRS2500 (1 μmol/L), were added to each organ bath before EFS to observe the contractile responses in the strips.

The strips were preincubated in a single concentration of BBH (40 μmol/L) to observe its effects on neurotransmitters such as L-NAME (10 μmol/L), α,β-MeATP (100 μmol/L), MRS2500 (1 μmol/L), and TTX (1 μmol/L) during spontaneous or EFS-induced colonic contraction, respectively. In this *in vitro* study, each strip was subjected to a 1-h equilibration before the next experiment.

### Statistical Analysis

Data are expressed as mean ± standard error of the mean (SEM). *p* < 0.05 was considered statistically significant, and *n* indicates the number of samples. Statistical analysis and curve fit were performed using GraphPad Prism (GraphPad Software, San Diego, CA, United States). Calculations were performed using SPSS 20.0 on the basis of the number of individual tissue segments. Non-pairwise comparisons were performed using Student’s *t*-test. ANOVA was used in testing three or more variables for statistical significance. Nonlinear and linear regression analyses were also utilized as needed.

## Results

### Establishment of the IBS-D Rat Model

**FIGURE 3:**
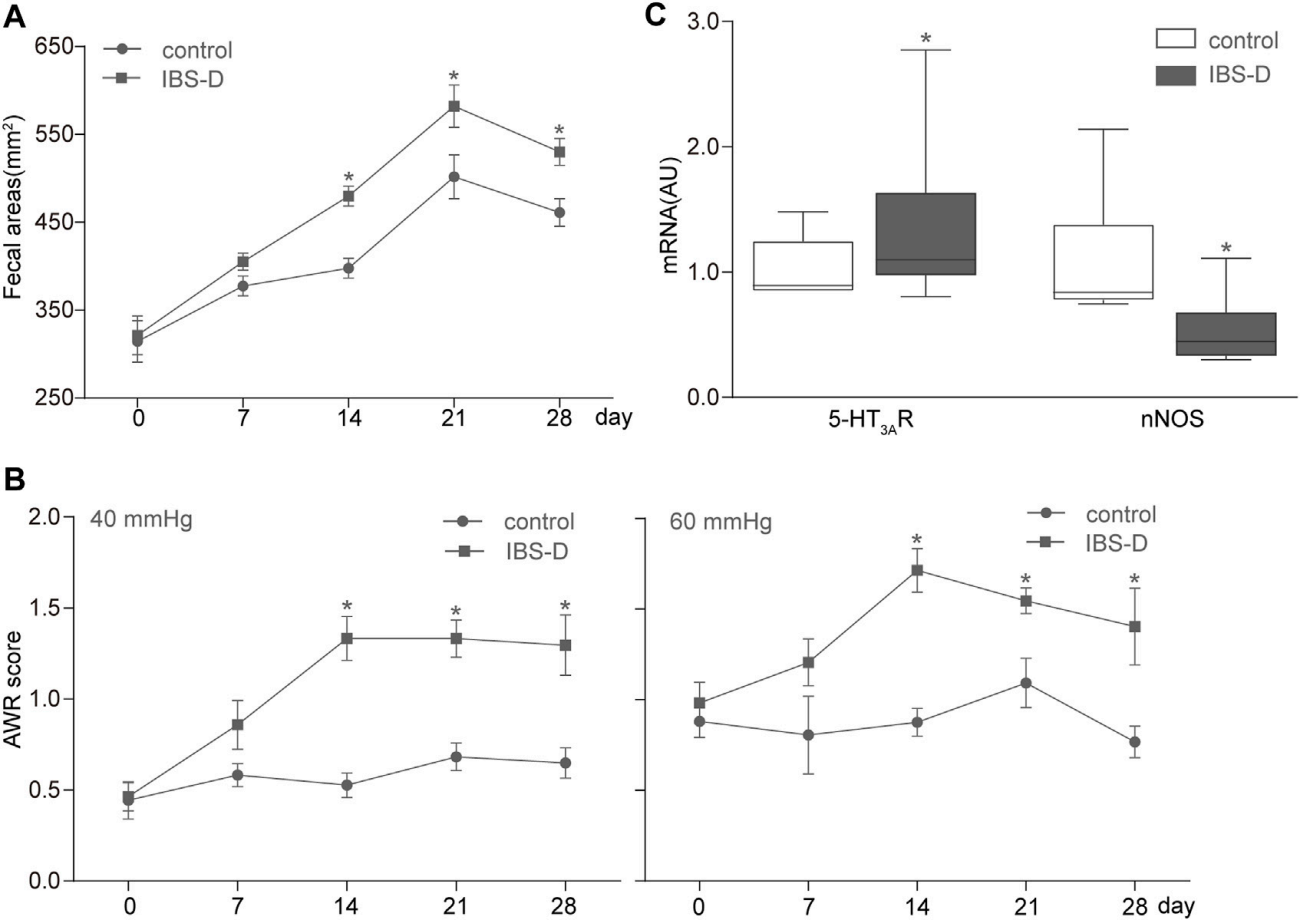
Establishment of the IBS-D rat model. **(A,B)** Measurement results of fecal areas and AWR scores at 40 and 60 mmHg on days 0, 7, 14, 21, and 28. **(C)** Box and whisker plots of the distribution and mean expression levels of 5-HT_3A_R and nNOS in the colonic tissues of the control and IBS-D groups. Data are represented as mean ± SEM. *n* = 6–12 rats per group. *denotes *p* < 0.05 compared with the control group.

We counted and measured the fecal areas of the control and IBS-D rats on days 0, 7, 14, 21, and 28. The fecal areas of the IBS-D group on days 14, 21, and 28 significantly increased (*p* < 0.05) compared with those of the control group (Figure 3A). Nevertheless, the fecal areas of both groups had no discrepancy on the 0th and seventh days. The change in 4-h fecal areas showed that the defecation frequency of the IBS-D group increased with the passage of time, which is consistent with the change in the bowel habit of clinical patients with IBS-D. This result indicated that an IBS-D rat model was successfully established by chronic restraint stress.

All rats were subjected to graded CRD (20, 40, 60, and 80 mmHg) and given AWR scores. The IBS-D group showed a remarkable increase in AWR compared with the control group (Figure 3B). These changes were substantial for all the intensities of CRD; hence, the applied chronic restraint stress successfully established the IBS-D rat model and resulted in a visceral hypersensitivity similar to that of humans.

Furthermore, qRT-PCR analysis of mRNA levels showed that the expression of 5-HT_3A_R increased, whereas that of nNOS decreased in the IBS-D group compared with the control group (Figure 3C).

### BBH Ameliorated Symptoms in IBS-D Rats

**FIGURE 4:**
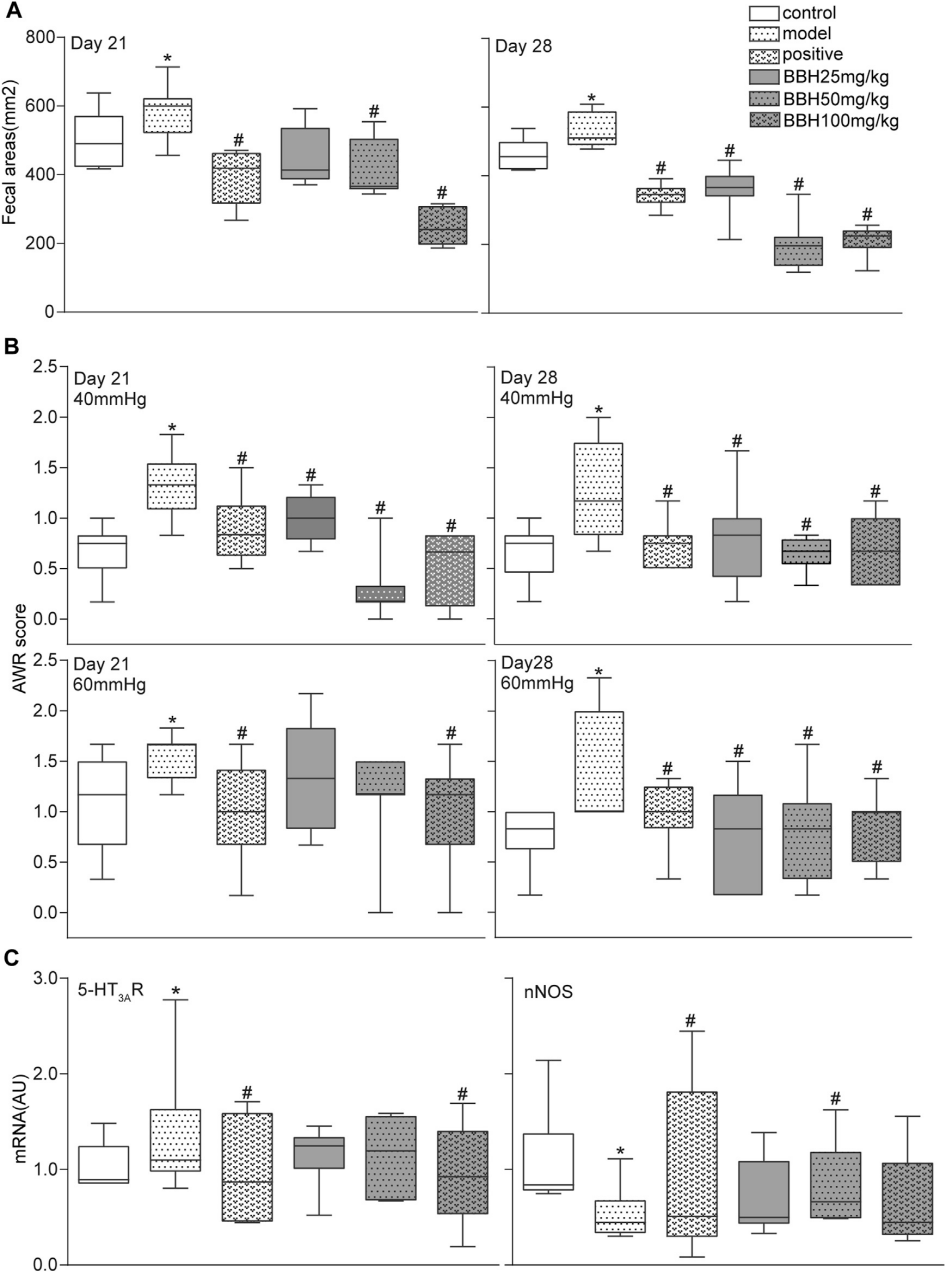
BBH ameliorated symptoms in IBS-D rats. Box and whisker plots of the distribution and mean of fecal areas, AWR scores, and the expression levels of 5-HT_3A_R and nNOS of the control group, model groups, and four treatment groups on days 21 and 28. The AWR scores were measured in response to graded CRD (40 and 60 mmHg). *n* = 6–12 rats per group. ^*^denotes *p* < 0.05 compared with the control group. ^#^denotes *p* < 0.05 compared with the model group.

The 4-h fecal areas and AWR scores (under 40 and 60 mmHg CRD) of the IBS-D group significantly increased (*p* < 0.05 or *p* < 0.01) on days 21 and 28 compared with the control group. This finding indicated the presence of increased defecation frequency and visceral hypersensitivity, which were consistent with the characteristics of hyperalgesia in clinical patients with IBS-D. Furthermore, the fecal areas and AWR scores of the positive drug group (30 mg/kg pinaverium bromide) and BBH groups (50 and 100 mg/kg) decreased obviously (*p* < 0.05 or *p* < 0.01) compared with the model group (no medication). In brief, 50 and 100 mg kg^−1^ BBH considerably reduced the 4-h fecal areas and improved the high sensitivity of visceral neurons. Although the AWR scores of the (25 mg/kg) BBH group decreased significantly compared with the model group (*p* < 0.05), no obvious change in fecal areas was observed (*p* > 0.05). Figures 4A,B only show the data of fecal areas and AWR scores at 40 and 60 mmHg CRD on days 21 and 28.

The results of qRT-PCR analysis showed that chronic restraint stress changed the colonic mRNA expression levels of 5-HT_3A_R and nNOS in the model group compared with the control group. However, the LC8 and SLC17A9 mRNA expression in the model and control groups had no statistical difference. Pinaverium bromide (30 mg/kg) treatment inhibited the mRNA expression of 5-HT_3A_R and nNOS in the model group. qRT-PCR analysis of mRNA levels in BBH-treated rats showed similar effects as pinaverium bromide. BBH treatment led to the downregulation of 5-HT_3A_R mRNA level and the upregulation of nNOS level in the IBS-D group (Figure 4C). BBH (25, 50, and 100 mg/kg) had no effect on LC8 and SLC17A9 mRNA expression in the GI tract of rats. These changes show the treatment effects of BBH. BBH may improve the symptoms of visceral hypersensitivity and abnormal defecation frequency in the IBS-D group by regulating the mRNA expression levels of 5-HT_3A_R and nNOS to achieve a treatment effect.

### Effect of BBH on the Colonic Contractile Responses of Isolated Longitudinal Smooth Muscles

#### 3.1.1 BBH Inhibited the Spontaneous Contraction of Isolated Colonic Longitudinal Smooth Muscles

**FIGURE 5:**
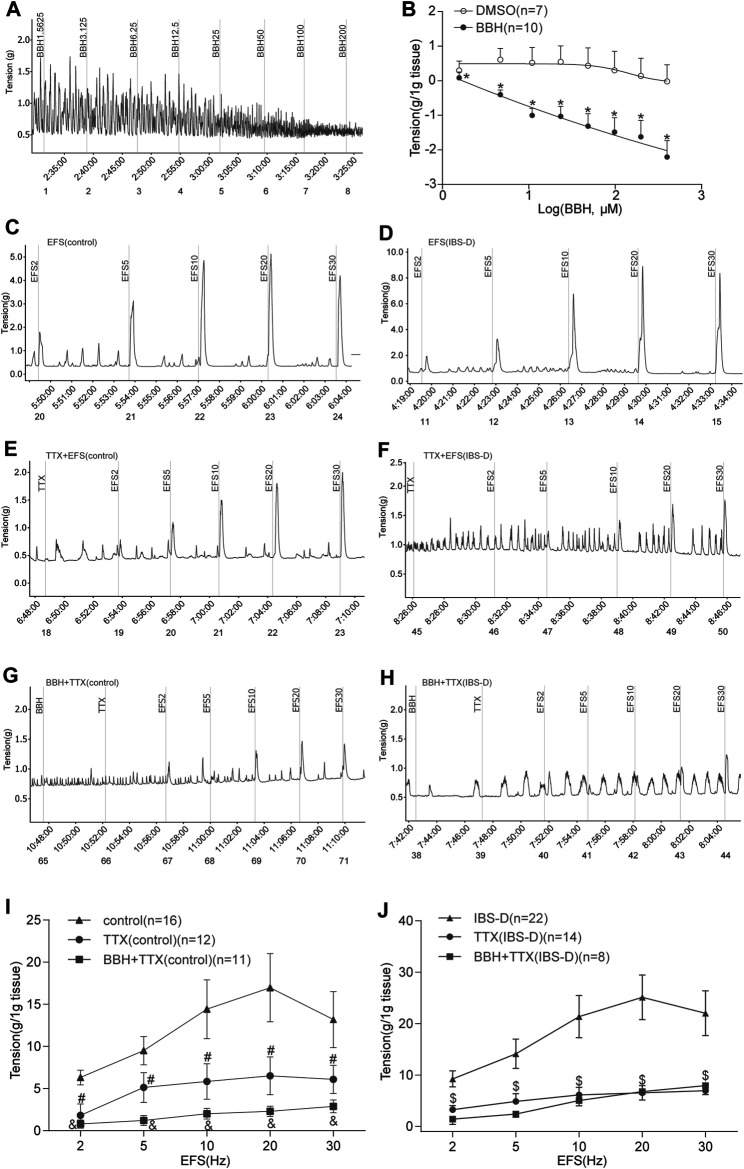
BBH inhibited spontaneous and EFS-induced contractions of TTX-treated isolated colonic longitudinal smooth muscle. **(A,B)** Mechanical recording and linear regression curve of the cumulative log concentration–response of the effect of BBH (1.5625–200 μmol L^−1^) on the spontaneous contractions of the isolated colonic longitudinal smooth muscle. The EC_50_ of BBH was 25.17 μmol/L. DMSO had no effect. *denotes *p* < 0.01 compared with the vehicle DMSO group. BBH: *n* = 10, DMSO: *n* = 7. **(C–J)** Mechanical recording **(C–H)** and histogram **(I–J)** of the effect of TTX and BBH + TTX treatments on the EFS-induced contraction response of colonic longitudinal smooth muscle in the control and IBS-D groups. **(C)**: EFS-induced control group (*n* = 16). **(D)**: EFS-induced IBS-D group (*n* = 22). **(E)**: TTX-treated control group (*n* = 12). **(F)**: TTX-treated model group (*n* = 14). **(G)**: BBH + TTX-treated control group (*n* = 11). **(H)**: BBH + TTX-treated IBS-D group (*n* = 8). **(I)**: BBH inhibited EFS-induced contractions of TTX-treated colonic tissues in the control group. # denotes *p* < 0.05 compared with the EFS-induced control group & denotes *p* < 0.05 compared with the TTX-treated control group. **(J)**: BBH inhibited the EFS-induced contractions of TTX-treated colonic tissues in the IBS-D group. $ denotes *p* > 0.05 compared with the EFS-induced IBS-D group. Error bars represent mean ± SEM.

Colonic longitudinal smooth muscle strips were prepared in an organ bath, and the basic tension of spontaneous contraction occurred within a few minutes. The addition of seven cumulative concentrations of BBH (1.5625, 3.125, 6.25, 12.5, 25, 50, 100, and 200 μmol/L) successively led to a decrease in the tension of colonic longitudinal muscles in a concentration-dependent manner (*p* < 0.01). Meanwhile, DMSO did not affect the strips. The EC_50_ of BBH was 25.17 μmol/L. Thus, the BBH concentration of 40 μmol/L was used in further experiments (Figures 5A,B).

#### 3.1.2 BBH Inhibited the EFS-Induced Contraction of the TTX-Treated Isolated Colonic Longitudinal Smooth Muscles

The intestinal nerve activated by EFS can simulate the contraction produced by various nerve cells *in vivo*; thus, it was used to observe neurogenic contraction response. We explored the effect of BBH on the contraction of isolated longitudinal colonic smooth muscle under TTX. The tissue was preincubated with BBH (40 μmol/L) for 3 min. TTX (1 μmol/L) was added to the organ bath to evaluate the contractile response. EFS was induced using 2–30 Hz for 10 s. The TTX-treated contractions and EFS-induced tissues in the control and IBS-D groups had a significant difference (*p* < 0.05) in contractile responses; thus, the contractile responses to EFS were mediated *via* neural stimulation (Figures 5C–F,I,J). The results of BBH + TTX-treated tissues in the IBS-D group showed no significant differences compared with those of TTX-treated tissues (*p* > 0.05). Nevertheless, the effect of the diastole produced by TTX preincubated with BBH was decreased compared with that produced by TTX only in the control group (*p* < 0.05; Figures 5E–J).

#### 3.1.3 BBH Inhibited the Cholinergic Contractile Responses of Isolated Colonic Longitudinal Smooth Muscles

**FIGURE 6:**
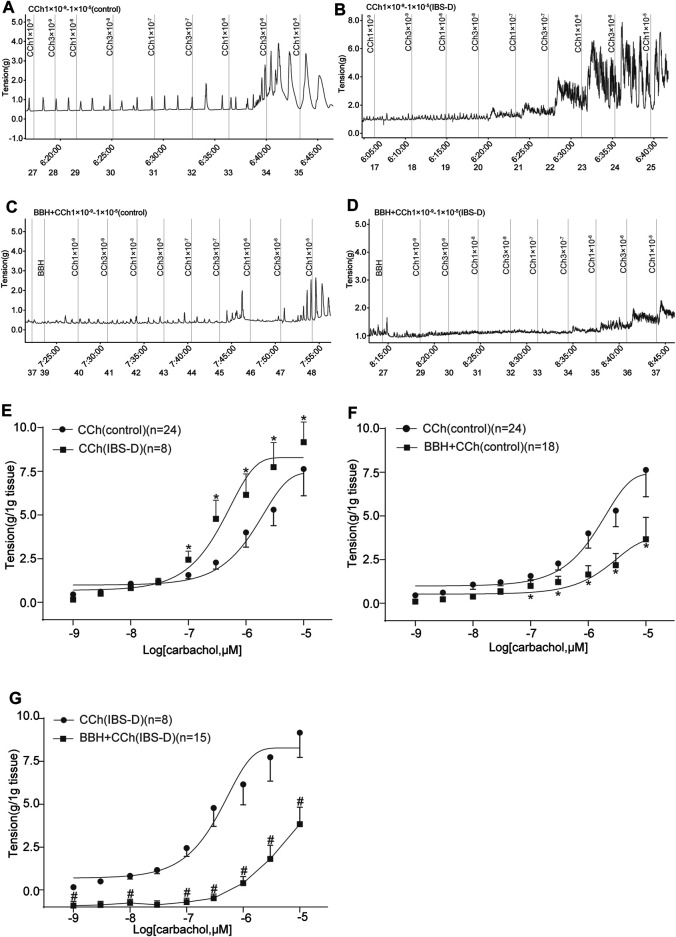
BBH inhibited the cholinergic contractile responses of isolated colonic longitudinal smooth muscles. Mechanical recording **(A–D)** and cumulative log concentration–response curve **(E–G)** of the effect of CCh and BBH + CCh treatments on the contractions of isolated colonic longitudinal smooth muscles in the control and IBS-D groups. **(A)**: CCh-induced control group (*n* = 24). **(B)**: BBH + CCh-induced control group (*n* = 18). **(C)**: CCh-induced IBS-D group (*n* = 8). **(D)**: BBH + CCh-induced IBS-D group (*n* = 15). **(E)**: Cholinergic contractile responses in the control and IBS-D groups. The EC_50_ obtained in the IBS-D group was 0.4906 μmol/L, whereas that in the control group was 2.527 μmol/L * denotes *p* < 0.05 compared with the CCh-induced control group. **(F)**: BBH inhibited the cholinergic contractile responses in the control group. * denotes *p* < 0.05 compared with the CCh-induced control group. **(G)**: BBH inhibited the cholinergic contractile responses in the IBS-D group. # denotes *p* < 0.05 compared with the CCh-induced IBS-D group. Error bars represent mean ± SEM.

In previous studies, we showed that the contractile responses of longitudinal smooth muscle by cholinergic neurotransmission are inhibited in the jejunum but excited in the colon of IBS-D rat model induced by chronic restrain stress. The IBS-D group had higher (*p* < 0.05) contractile tension to the movement of isolated colon induced by CCh (1 × 10^−7^–1 × 10^–5^ mol/L) compared with the control group (Figures 6A,B,E). The EC_50_ obtained in the IBS-D group (0.4906 μmol/L) in the presence of CCh was relatively lower than that in the control group (2.527 μmol/L) as shown in Figure 6E. Moreover, pretreatment with 40 μmol/L BBH for 10 min could eliminate the colonic contraction responses caused by CCh in the control and IBS-D groups. The contractile tensions of BBH + CCh-treated colonic tissues in the control group were significantly lower (*p* < 0.05) than those of CCh-treated tissues (1 × 10^−7^–1 × 10^–5^ mol/L) (Figures 6A,C,F). Preincubation with 40 μmol/L BBH could significantly reduce the contractile tension of CCh-treated (1 × 10^−9^–1 × 10^–5^ mol/L) colonic tissue compared with the IBS-D group treated with CCh alone (*p* < 0.05 or *p* < 0.01; Figures 6B,D,G). Furthermore, the comparison of the colonic contractile responses of the control and IBS-D groups treated with BBH and CCh showed that the tension of the IBS-D group was much lower than that of the control group. In brief, the antagonistic effect of BBH on colonic smooth muscle was more obvious in the IBS-D group than the control group (Figures 6C,D,F,G).

#### 3.1.4 BBH Inhibited the EFS-Induced Contractions Elicited in the Presence of NO Blockade of Isolated Colonic Longitudinal Smooth Muscles

**FIGURE 7:**
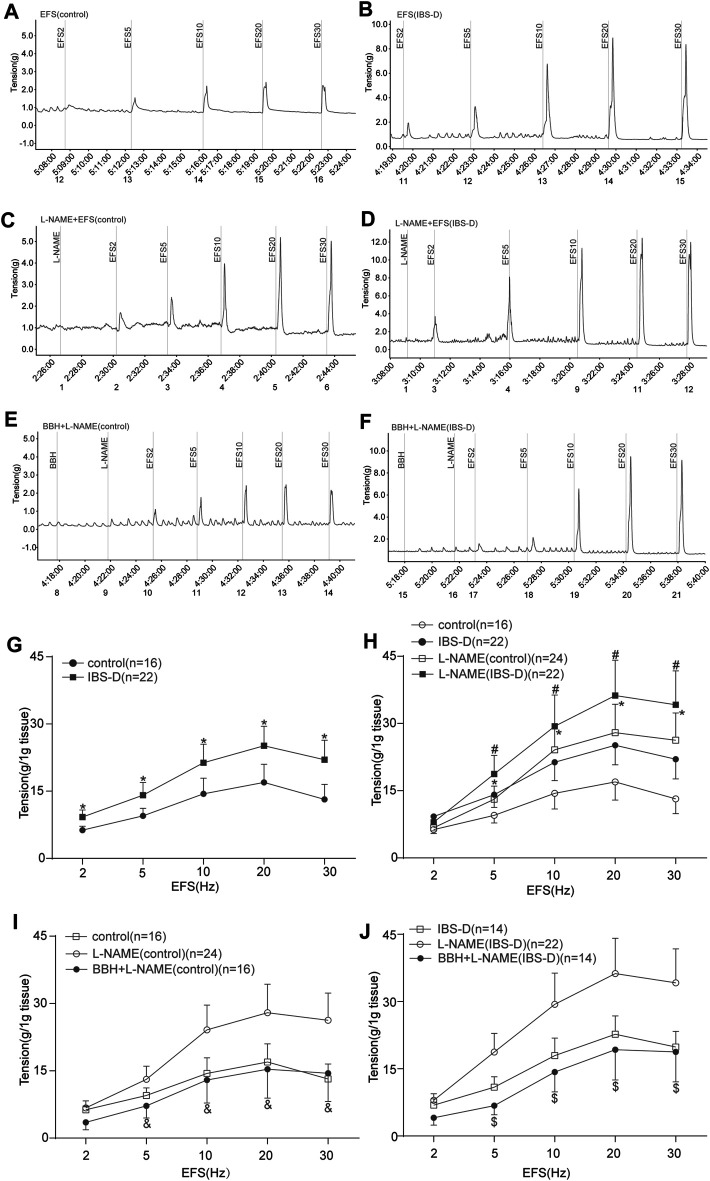
BBH inhibited the EFS-induced nitrergic contractile responses of isolated colonic longitudinal smooth muscles. Mechanical recording **(A–F)** and line chart **(G–J)** of the effect of L-NAME and BBH + L-NAME treatments on the EFS-induced contraction response of colonic longitudinal smooth muscles in the control and IBS-D groups. **(A)**: EFS-induced control group (*n* = 16) **(B)**: EFS-induced IBS-D group (*n* = 22) **(C)**: L-NAME-induced control group (*n* = 24). **(D)**: L-NAME-induced IBS-D group (*n* = 22). **(E)**: BBH + L-NAME-induced control group (*n* = 16). **(F)**: BBH + L-NAME-induced IBS-D group (*n* = 14). **(G)**: EFS-induced contractile responses in the control and IBS-D groups. * denotes *p* < 0.05 compared with the EFS-induced control group. **(H)**: EFS-induced nitrergic contractile responses in the control and IBS-D groups. * denotes *p* < 0.05 compared with the EFS-induced control group; # denotes *p* < 0.05 compared with the EFS-induced IBS-D group. **(I)**: BBH inhibited the EFS-induced nitrergic contractile responses in the control groups & denotes *p* < 0.05 compared with the L-NAME-induced control group. **(J)**: BBH inhibited the EFS-induced nitrergic contractile responses in the IBS-D groups. $ denotes *p* < 0.05 compared with the L-NAME-induced IBS-D group. Error bars represent mean ± SEM.

The contractile responses of EFS-induced colon tissues of the IBS-D group increased significantly compared with those of the control group (*p* < 0.05; Figures 7A,B,G). The contraction caused by EFS after preincubation with L-NAME (10 μmol/L) was higher (*p* < 0.05) than that by EFS induction only in the control and IBS-D groups (Figures 7A–D,H). Colonic tissues in the control group pretreated with L-NAME showed a remarkably lower EFS-induced contraction than those in the IBS-D group at 5–30 Hz (Figures 7C,D,H). Preincubation with BBH (40 μmol/L for 10 min) significantly reduced the combined contraction stimulated by L-NAME and EFS (5–30 Hz, 10 s) in the control and IBS-D groups (*p* < 0.05) and had a remarkable impact on the IBS-D group (Figures 7C–F,I,J).

#### 3.1.5 BBH Inhibited the EFS-Induced Contractile Responses of Isolated Colonic Longitudinal Smooth Muscles Treated With P2Y_1_ Receptor Antagonist

**FIGURE 8:**
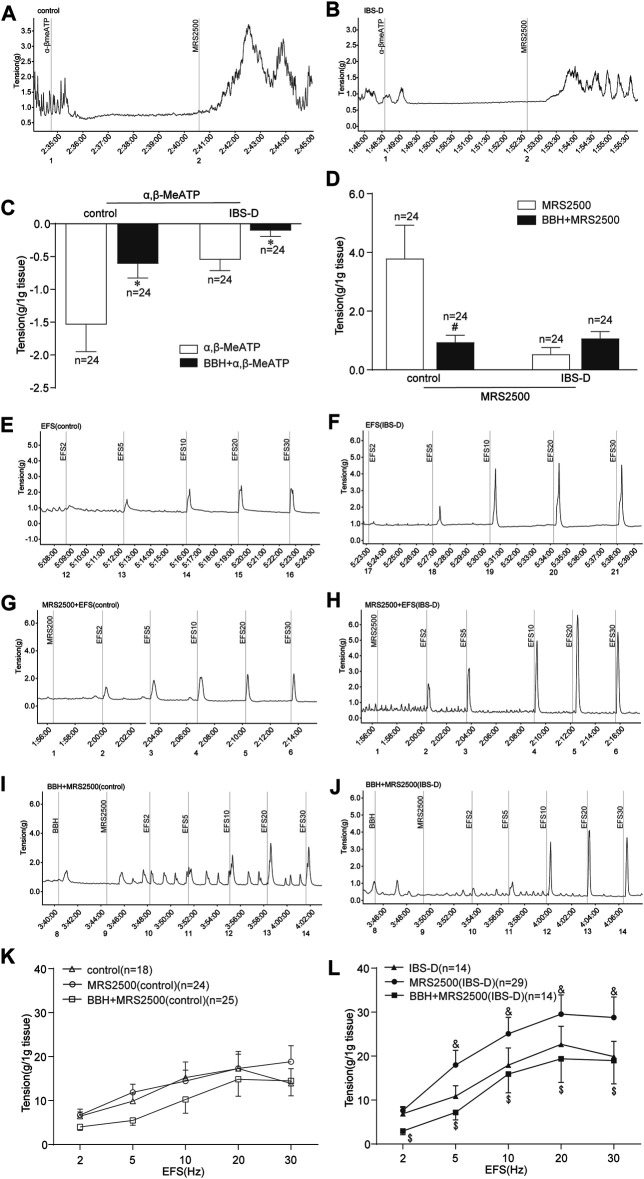
BBH inhibited the EFS-induced contractile responses of isolated colonic longitudinal smooth muscles treated with P2Y_1_ receptor antagonist. Mechanical recording **(A, B, and E–J)**, histogram **(C,D)**, and line chart **(K,L)** of the inhibitory effect of BBH on the tension of the α,β-MeATP-treated and MRS2500-treated isolated colonic longitudinal smooth muscles in the control and IBS-D groups. **(A)**: α,β-MeATP-treated (*n* = 24) and MRS2500-treated (*n* = 24) control groups. **(B)**: α,β-MeATP-treated (*n* = 24) and MRS2500-treated (*n* = 24) IBS-D groups. **(C)**: *denotes *p* < 0.05 compared with the α,β-MeATP-treated group. **(D)**: # denotes *p* < 0.05 compared with the MRS2500-treated control group. **(E)** EFS-induced control group (*n* = 18). **(F)**: IBS-D group (*n* = 14). **(G)**: EFS + MRS2500-treated control group (*n* = 24). **(H)**: EFS + MRS2500-treated IBS-D group (*n* = 29). **(I)**: BBH + MRS2500-treated and EFS-induced control group (*n* = 25). **(J)**: BBH + MRS2500-treated and EFS-induced IBS-D group (*n* = 14). **(K)**: Inhibitory effect of BBH on the MRS2500-treated control group. **(L)**: Inhibitory effect of BBH on the MRS2500-treated IBS-D group & denotes *p* < 0.05 compared with the EFS-induced IBS-D group. $ denotes *p* < 0.05 compared with the EFS + MRS2500-induced IBS-D group. Error bars represent mean ± SEM.

α,β-MeATP (100 μmol/L) was added to the organ bath, and MRS2500 (1 μmol/L) was added 2 min later. Then, changes in the tension of colonic tissues without EFS were observed. α,β-MeATP could inhibit the spontaneous contraction of colonic smooth muscle in the control and IBS-D groups. Meanwhile, this relaxing effect on the colon could be antagonized by BBH. The results showed significant differences between the BBH- and α,β-MeATP-treated control group, and similar results were observed in the BBH- and α,β-MeATP-treated IBS-D group (*p* < 0.05; Figures 8A–C). Indeed, the inhibitory effect of α,β-MeATP on colonic spontaneous contraction could be completely antagonized by the addition of MRS2500. Significant differences were observed between the BBH- and MRS2500-treated control groups (*p* < 0.05), whereas no significant difference (*p* > 0.05) was observed between the BBH- and MRS2500-treated IBS-D groups (Figures 8A,B,D). In addition, EFS-induced (2–30 Hz, 10 s) contractions were significantly increased in the MRS2500-treated IBS-D group compared with the untreated IBS-D group (*p* < 0.05; Figures 8F,H,L). The contractions in the MRS2500-treated IBS-D group were significantly decreased by the co-application of BBH (40 μmol/L) at the presented EFS compared with that in the IBS-D group treated with MRS2500 only (*p* < 0.05; Figures 8H,J,L). By contrast, the comparison between MRS2500-treated contractions and EFS-induced contractions had no substantial differences in the control group, and BBH + MRS2500-treated contractions and MRS2500-induced contractions also had no remarkable differences (Figures 8E,G,I,K).

## Discussion

The etiology of IBS-D is complicated, and its physiological and pathological mechanisms have not been elucidated yet . Current drug treatment alleviates symptoms but has limited effect. Therefore, a safe, effective, and targeted drug for IBS-D is urgently needed.

BBH is a derivative of berberine, which is the major constituent of *Rhizoma coptidis*, a herbal constituent used to treat IBS. Previous studies have shown that BBH has potential anti-inflammatory, antibacterial, analgesic, and antidiarrheal effects and a wide range of biological activities, especially in regulating the release of some neurotransmitters, which are involved in the pathological process of various neurological diseases. A recent study (Yu et al., 2019) has shown that intestinal immune disorders are closely related to the pathogenesis of IBS. Cytokines are a part of intestinal immune regulation. Intestinal inflammation leads to abdominal pain, which is closely related to the nuclear factor kappa-B signaling pathway in the immune response. BBH can inhibit intestinal mucosal inflammation in IBS rats by affecting the signal pathway, thus reducing intestinal motility and visceral hypersensitivity. Berberine can significantly reduce the levels of pro-inflammatory cytokines (TNF, IFN-γ, KC, IL-7) in colon tissue and improve the symptoms of intestinal injury and colitis model induced by dextran sulfate sodium in mice (Yan et al., 2012). Berberine can inhibit the immunoreaction caused by T helper (Th)1, Th2, and Th17 to improve the inflammatory symptoms of ulcerative colitis. On the other hand, it can reduce the expression of zonula occludens-1 protein related to tight junction (TJ) protein in the colon by inhibiting Th17 reaction (Li et al., 2016). TJ protein is a component of the epithelial barrier that prevents GI flora from affecting the environment of luminal epithelial tissue. These findings suggest that the therapeutic potential of berberine against IBD is partially realized by improving the intestinal microenvironment.

However, IBS is a functional bowel disease characterized by abdominal pain with changes in defecation habits or abnormal fecal characteristics. The degree of pain or discomfort in patients with IBS is related to the high sensitivity of the colon and rectum. Research on pain (Gold and Gebhart, 2010) suggests that continuous peripheral afferent stimulation is a necessary and sufficient condition for chronic pain, and cutting off afferent stimulation can effectively relieve pain and discomfort. One of the best examples is that local rectal anesthesia can quickly relieve abdominal pain, discomfort, and tenderness in IBS (Feng et al., 2012). Another experiment conducted on a human colon biopsy showed that intestinal mediators can induce increased visceral sensitivity (Balestra et al., 2012). We paid attention to this point in a previous study (Zhou R. T. et al., 2018). Tissue samples were taken from the antrum, duodenum, middle ileum, and colon of the modified stress-induced model rats, and then sectioned and stained chemically. The results showed that compared with the control group, no obvious pathological changes were observed in the tissue samples of the model, and organic lesions could be basically excluded. In the preparation of visceral hypersensitivity animal model, colonic injection of sodium butyrate solution (110 mg/ml) twice a day for 3 days resulted in non-inflammatory visceral hyperalgesia in rats (Lian et al., 2010). In addition, almost no GI inflammatory response were observed in IBS mice induced by *Trichinella spiralis* infection, but the intestinal hypersensitivity persisted (Wang et al., 2014). Therefore, the primary focus of our exploration in IBS-D rat model and the pharmacological mechanism of BBH is not inflammation, but the regulation of intestinal nervous system homeostasis.

GI dysfunction in IBS-D is closely associated with the colon (Kanazawa et al., 2008; Manabe et al., 2010). Our previous studies confirmed that the abnormal neurotransmission function in the IBS-D rat model occurs in the colon rather than in the jejunum. Increased defecation frequency is one of the diagnostic criteria that can distinguish IBS-D from the other IBS subtypes, and the abnormal GI motility function of IBS-D is often associated with high visceral sensitivity. The purpose of this work was to provide insights into the treatment effects and mechanisms of BBH in an IBS-D rat model. The major finding of this study is that chronic restraint stress results in a remarkable alteration in defecation frequency, visceral hypersensitivity, and intestinal neurotransmission in IBS-D rats. These stress-induced changes in intestinal function in rats, which indicate a homeostasis imbalance in the ENS, are similar to stress-induced changes in intestinal motility in humans. We found that BBH exerted a therapeutic effect on stress-induced IBS-D rats by inhibiting neurotransmission in colonic smooth muscle. The 4-h fecal area of the IBS-D group was remarkably different compared with that of the control group starting on the 14th day as shown in the Image-J analysis. The rats were then tested for behavioral responses to CRD. The behavioral effect of colon distention was recorded using a semiquantitative scale to measure AWR, which is a kind of unconscious motor reflex similar to visceral motor reflex (Al-Chaer et al., 2000), in response to a range of CRD intensities (20, 40, 60, and 80 mmHg). The results showed that the IBS-D group had substantially higher AWR scores compared with the control group. The changes were relevant for our modified rat model and indicated the successful establishment of the IBS-D rat model, which had increased defecation frequency and visceral hypersensitivity similar to those of humans, by chronic restraint stress. These changes in the model were also verified by the results of qRT-PCR analysis. The mRNA expression levels of neurotransmission-related transmitters or receptors, such as 5-HT_3A_R, were increased, whereas that of nNOS was decreased in the IBS-D group compared with the control group. Meanwhile, BBH could reverse this outcome and showed an effect similar to that of positive drugs. Thus, the increased defecation frequency and visceral hypersensitivity in the IBS-D model induced by restraint stress may be related to changes in GI tract neurotransmission, which lead to the imbalance of ENS homeostasis. This mechanism is perhaps important in the physiology and pathology of IBS-D. Moreover, BBH could reduce abnormal defecation frequency and improve visceral hypersensitivity in IBS-D rats probably by changing the colonic neurotransmission in the IBS-D model.

NO produced by nNOS is an inhibitory small molecular neurotransmitter. The absence or severe deficiency of nNOS can lead to the failure or weakening of nitrergic neurotransmission. LC8 is not only a part of the dynamic protease complex, but also has the activity of nNOS inhibitor. When it binds to nNOS, it can make the structure of nNOS dimer unstable and inactive, thus affecting the transmission of nitrergic nerves (Jaffrey and Snyder, 1996; Chaudhury et al., 2008). The neurotransmission of purine is related to ATP, while the transport of nucleotides in cells cannot cross the cell membrane freely, and its storage and transshipment must be transmembrane by means of vesicle (Burnstock, 2014). The protein encoded by human or mouse SLC17A9 gene is a vesicular nucleotide transporter capable of carrying nucleotides such as ATP, ADP, and GTP, so the novel technique of SLC17A9 staining can specifically identify purinergic vesicles of intestinal neuronal varicosity (Larsson et al., 2012; Hiasa et al., 2014). Once released, the stored ATP and nucleotide molecules in the vesicle bind to specific P2X ion channel receptors or P2Y metabolic receptors on the surface of vesicular secretory cells or adjacent cells and produce biological effects (Burnstock and Novak, 2013).

The results of our study showed that LC8 and SLC17A9 mRNA expression in the model and control groups had no statistical difference, and BBH (25, 50, and 100 mg/kg) had no effect on LC8 and SLC17A9 mRNA expression in the GI tract of rats. We speculate that this may be related to other targets of BBH acting on IBS-D. LC8 can not only connect with nNOS, but also interact with Myosin Va. LC8 is a transporter that connects nNOS and Myosin Va (Chaudhury et al., 2011). Myosin is one of the three superfamily molecular motors in eukaryotes, which participates in a wide range of cellular physiological processes and functions, such as intracellular substance transport, transcriptional regulation, signal transduction, cell movement, and smooth muscle contraction. Myosin Va is the most widely distributed in the nervous system of humans and mice (Hammer and Wagner, 2013). In DBA mice with Myosin Va gene deficiency, the expression of Myosin Va in nerve endings was significantly decreased or deleted, resulting in decreased purinergic inhibitory junction potential and nitrergic inhibitory junction potential (Chaudhury et al., 2011). The *in situ* proximity ligation assay (PLA) used to study protein–protein interaction showed that SLC17A9 and Myosin Va were simultaneously expressed in the neuronal varicosity. In DBA mice, the PLA signals of SLC17A9 and Myosin Va were missing, and the exocytosis-entry rate of the varicosity membrane was low, suggesting that Myosin Va plays an important role in the transport of purinergic vesicles to the varicosity membrane and exocytosis (Chaudhury et al., 2012). Our team has carried out a series of studies on the role of Myosin Va in GI neurotransmission and the mechanism of IBS-D. Combined with the above analysis, we believe that the regulatory mechanism of BBH on neurotransmitter release-related proteins such as nNOS, LC8, and SLC17A9 may depend on the regulation of Myosin Va.

Our further research proved that the isolated colonic neurotransmission of IBS-D model induced by chronic restraint stress changed, and BBH exerted a treatment effect on the IBS-D model by blocking the neurotransmission of acetylcholine (ACh) and nitrergic agents and by inhibiting P2Y_1_ receptor antagonist-induced contractile responses in the colonic smooth muscles of IBS-D rat.

The addition of TTX blocked the neurotransmission of ENS and remarkably decreased the contractile responses of colonic smooth muscles in the control and IBS-D groups compared with the non-TTX-treated group. TTX preincubated with BBH continued to inhibit these responses in normal rats but had no effect on the IBS-D group. It is possible that the responses of BBH found in the present study are TTX sensitive, showing that they are originated by neural-mediated activity. Therefore, what neurons and neurotransmitters are involved in this neural-mediated activity? We then made the following exploration.

Cholinergic neurons are not only the earliest species and ontogeny neurons, but also have the largest number in the ENS. As the main excitatory motor neurotransmitter, ACh has an excitatory effect on the GI tract. ACh receptors (AChRs) in the postsynaptic membrane convert the ACh signals released by motor neurons into endplate potentials to stimulate the action potential and contraction of muscle fibers. The main neurotransmitter in this reflex arch is ACh. The agonist of ACh, CCh (1 × 10^–9^ 1 × 10^–5^ mol/L), could considerably promote the contractile responses in colons of stress-induced IBS-D rats, which is consistent with published data (Guarino et al., 2017). Pretreatment with a single BBH concentration strongly inhibited the CCh-induced contractions in the IBS-D rat colon *in vitro*. Our study is the first to demonstrate that BBH exerts an inhibitory effect on CCh-induced colonic contraction. However, further research is necessary to determine whether this inhibitory effect against ACh operates on muscarinic receptors, nicotinic AChRs, or both.

L-NAME, a NOS inhibitor, can suppress the production of NO and inhibit the relaxation of smooth muscles and NANC relaxant responses to induce contraction. The present study (Suthamnatpong et al., 1993) reported that NO plays an important role in the relaxation of longitudinal muscle preparations from rat proximal colon. NO could activate calcium-activated and ATP-sensitive K^+^ channels in human colon strips (Sahin et al., 2001). K^+^ channels play an important role in the regulation of membrane excitability and tonus in various smooth muscle cells (Li et al., 1999). The activation of these channels causes smooth muscle membrane hyperpolarization, which leads to relaxation. By contrast, the inhibition of these channels produces membrane depolarization and smooth muscle contraction (Gil et al., 2010). The qRT-PCR analysis of mRNA levels showed that the expression of nNOS was decreased in stress-induced IBS-D rat colon compared with the control group, and BBH may upregulate the expression of nNOS in IBS-D. Stimulation with EFS *in vitro* showed that the colonic longitudinal muscles of IBS-D rats had higher contractile tension when incubated by L-NAME compared with the control group, and BBH could inhibit this contractile response. Overall, the obtained data indicated that the etiology of IBS-D may be caused by reduced nNOS expression and NO release. Therefore, the inhibitory effect of BBH on contraction may be caused by the promotion of nitrergic neurotransmission in the colonic longitudinal muscle of IBS-D rats. These results suggest that BBH may increase the synthesis of NO in the colon of rats. *In vitro* experiments showed that BBH could exert a diastolic effect through the nitrergic neurotransmission of IBS-D, indicating that BBH may be an NO donor.

In addition, our research showed that BBH exerts significant effects on the treatment of diarrhea. Abnormal ion transport in the intestinal tract is closely related to the occurrence of diarrhea (Coté and Buchman, 2006). Secretory diarrhea refers to diarrhea caused by excessive secretion of water and electrolytes in intestinal mucosa crypts beyond the absorption capacity of intestinal villous epithelial cells (Schiller, 1999). Different types of ion channels are present on the intestinal cell membrane, such as sodium–potassium pump, calcium channel, chloride channel, and sodium–hydrogen exchanger. Changes in cellular second messengers such as cAMP, cGMP, and calcium-related transport channels; hormone secretion from endocrine tumors, bile salts, and long-chain fatty acids; and inflammatory mediators may stimulate mucosal cells and lead to secretory diarrhea (Field, 2003). cAMP can increase the probability of opening low conductance chloride channels and promote more chloride ion transport to the extracellular space; at the same time, cAMP can also enhance the activity of sodium and potassium dichloride cotransporter in the basement membrane and promote more sodium and potassium pump and potassium channel plasma transporters to enter the top membrane of intestinal epithelial cells, resulting in diarrhea caused by the destruction of electrolyte absorption balance of intestinal epithelial cells (Tabcharani et al., 1991). The opening of cAMP-activated potassium channels on the basement membrane can promote the repolarization of cells, counteract the depolarization effect caused by the opening of chloride channels in the basement membrane, and make chloride ions secrete into the intestinal cavity. The increase of chloride ion content promotes the secretion of intestinal electrolytes, leading to diarrhea (Kunzelmann et al., 2001). Studies on the antisecretory effects of berberine in rat ileum have been reported, and the alkaloid berberine may inhibit intestinal ion secretion and mucosal adenylate cyclase and Na-K-ATPase activities *in vitro* (Tai et al., 1981). Evidence provided in a previous study (Taylor et al., 1999)shows that secretory diarrhea is also modulated by the ENS, and berberine inhibits ion transport in human colonic epithelia. Data suggest the inhibition of basolateral K^+^ conductance on epithelial cells as the mechanism of action of this antidiarrheal drug. However, further studies are needed to confirm whether its antidiarrheal mechanism is related to its inhibition of intestinal secretion in IBS-D rats.

Purinergic neurotransmitters are responsible for NANC inhibitory responses in the GI tract. MRS2500 is considered the most potent P2Y_1_ antagonist and has been proven to be inactive on other purine receptors such as P2X, P2Y_12_, and P2Y_13_. α,β-MeATP is an unselective P2X receptor agonist (Alexander et al., 1999). A study indicated that α,β-MeATP, a stable analog of ATP, mimics endogenous purinergic mediator and causes the inhibition of spontaneous contractions in human and rat colons (Martínez-Cutillas et al., 2014). Our study showed that α,β-MeATP could inhibit the spontaneous contraction of colonic smooth muscle in the control and IBS-D groups in the absence of EFS induction. This relaxing effect on the colon could be antagonized by BBH. In addition, the inhibitory effect of α,β-MeATP on colonic spontaneous contraction could be completely antagonized by the addition of MRS2500. This is consistent with the results of previous studies (Martínez-Cutillas et al., 2014). The spontaneous contractile responses of colonic smooth muscle were restored in the control and IBS-D groups by BBH, and the effect of MRS2500 in the control group could be inhibited by BBH (had no effect on the IBS-D group). Interestingly, MRS2500 produced a greater contractile effect than EFS induction, and BBH inhibited these responses but only in the IBS-D group. These results indicated that P2Y_1_ receptors participate in the regulation of ENS and the coordination of intestinal movement in rats and play an important role in colonic motility disorders in IBS-D rats. Therefore, BBH acts on the P2Y_1_ receptor to treat IBS-D. Thus, the mechanism of BBH action was mediated by purinergic neurotransmission.

In general, the differences in the colon of IBS-D rat model from that of normal animal were as follows: 1) The mRNA expression of 5-HT receptors was activated, whereas that of nNOS was decreased. 2) Cholinergic receptors were activated, and colon contraction was promoted. 3) NOS was inhibited, NO release was decreased, and diastolic effect was inhibited. 4) P2Y_1_ receptor was activated and impeded the diastolic effect. The GI tract contractile responses of IBS-D rats can be improved by BBH by inhibiting the effect exerted by cholinergic, nitrergic, and purinergic neurotransmitters. In this work, neuropharmacological studies using GI tissue samples may help develop novel agents that target receptors that control smooth muscle contractility. However, additional studies are still needed to confirm the potential pharmacological target protein for BBH in the treatment of IBS-D.

## Data Availability

The raw data supporting the conclusions of this article will be made available by the authors, without undue reservation.

## Abbreviations

BBH, berberine hydrochloride; IBS-D, diarrhea-predominant irritable bowel syndrome; GI, gastrointestinal; CRD, colorectal dilation; AWR, abdominal withdrawal reflex; qRT-PCR, quantitative real-time polymerase chain reaction; EFS, electrical -field stimulation; L-NAME, Nω-nitro-L-arginine methyl ester hydrochloride; MRS2500, (1R*,2S*)-4-[2-iodo-6-(methylamino)-9H-purin-9-yl]-2-(phosphonooxy) bicyclo[3.1.0]hexane-1-methanol dihydrogen phosphate ester tetraammonium salt; α,β-MeATP, α, β-methyleneadenosine 5′-triphosphate trisodium salt; CCh, carbachol
